# A multispecies microcosm to assess the effect of footwear wastes on soil biota: a contribution towards sustainability

**DOI:** 10.1007/s11356-025-36824-3

**Published:** 2025-08-02

**Authors:** Beatriz Fernandes, Verónica Nogueira, Sirine Bouguerra, Cristiano Soares, Fernanda Fidalgo, Joana Machado, Susana M. P. Carvalho, Maria José Ferreira, Vera Pinto, José Rodrigues, Carlos M. Pereira, Ruth Pereira

**Affiliations:** 1 GreenUPorto/INOV4Agro, Campus de Vairão, Rua da Agrária 747, 4485 646 Vairão, Vila Do Conde Portugal; 2 Departamento de Biologia, Faculdade de Ciências, Universidade do Porto, Rua Do Campo Alegre S/N, 4169-007 Porto, Portugal; 3 Centro Tecnológico de Calçado de Portugal (CTCP), Rua de Fundões - Devesa Velha, 3700-121 São João da Madeira, Portugal; 4 CIQUP / Instituto de Ciências Moleculares (IMS), Departamento de Química E Bioquímica, Faculdade de Ciências, Universidade Do Porto, Rua Do Campo Alegre, 687, 4169-007 Porto, Portugal

**Keywords:** Chromium, Environmental assessment, Leather waste, Plant–soil interactions, Soil organisms, Soil enzymes

## Abstract

Due to the high quantities of solid waste with high concentrations of chromium that the footwear industry produces and its disposal sites, it is vital to understand whether leather residue itself is harmful to the ecosystem. Thus, a microcosm test with multispecies (*Brassica oleracea* and *Eisenia fetida*) was carried out using an agricultural soil contaminated with two different leather residues (Wet Blue and Finished Leather) from the footwear industry. After the stabilization period, *Brassica oleracea* seedlings and *Eisenia fetida* adults were exposed to these treatments. At the end of the experiment, a series of parameters were analysed in the *B. oleracea* leaves (leaf chlorophyl content, gas exchange measurements and photosynthetic parameters), in the *E. fetida* organisms (alkaline comet assay and biomarkers such as acetylcholinesterase and lipid peroxidation) and in the soils (total chromium content, enzymes activity and nitrogen mineralization and potential nitrification). In the case of soil’s enzymatic activity, even though some were significantly altered, no negative effects could be attributed to the leather residues. Moreover, the addition of residues to the soil did not significantly affect the plant species; however, the same was not observed for the earthworm *E. fetida* when in contact with Finished Leather. Overall, Finished Leather residue was the one that caused more effects on the parameters analysed and therefore its disposal should be carefully examined.

## Introduction

Since 2010, the global production of footwear has increased 21.2% making it an economically important industry worldwide (APICCAPS 2020). In 2021, the world footwear production increased by 8.6%, exceeding 22 billion pairs, being Asia responsible for 88.2% of production, followed by South America (4.7%), Africa (2.9%), Europe (2.8%) and North America (1.4%) (APICCAPS 2022). In the specific case of Portugal, the footwear industry is very important to the national economy as it employs over 40,000 people, in roughly 1600 companies. In 2022, a new record of production (€2.3 billion) and exports (more than €2 billion) was reached by the Portuguese footwear industry being the exportation responsible for more than 90% of production (APICCAPS 2023). In terms of leather footwear, Portugal has a strong specialization, which accounts for 86.2% of its footwear exports, being the 9th largest exporter and ranking among the 20 largest footwear exporters in the world (APICCAPS 2022). As the population and market demand increases, so does the waste generated during manufacturing and after use which is about 1.2 million tons per year, of which it is estimated that only 5% is recycled, 15% is reused and 80% is landfilled (CTCP 2012; European Commission 2020). In average, a shoe is manufactured using approximately 40 different materials such as synthetic materials, rubber, textiles and leather, of which leather being the main component, is responsible for generating around 1 × 10^5^ to 2 × 10^5^ tons of waste per year in Europe (CTCP 2012; Van Rensburg et al. 2020). The fact that a single shoe is composed by a complex mixture of materials is one of the primary reasons that this industry has a considerable environmental impact.

Leather, a versatile material appreciated for its durability and aesthetics, plays a vital role in footwear production. This transformation begins with animal hides or skins, which undergo a tanning process. Tanning stabilizes the raw hides, preventing putrefaction and enhancing their properties (Samidurai et al. 2022). This process significantly increases water resistance, reduces swelling and alters the material’s feel and appearance (Concord 2023). Several tanning methods exist, categorized as either mineral or organic tanning. The leather industry primarily relies on three processes: chrome, vegetable and aldehyde tanning. Each method imparts distinct characteristics to the final leather, influencing its softness, hardness, tightness and stretch (Monira and Mostafa 2023).

Currently, chrome tanning is the most used process, using chromium salts to achieve a lightweight, cost-effective leather with excellent heat and bacterial resistance (this leather is called wet-blue due to the blue colour that leather acquires when chromium salts are used (Suresh et al. 2001)). Leather production is a complex, time-intensive process involving multiple stages and various chemicals. The process can be broadly divided into three phases: (1) beamhouse, prepares the hides or skins for tanning by removing impurities and unwanted tissues; (2) tanning, transforms the hides into stable leather through the chosen tanning method; and (3) finishing (post-tanning), applies dyes, oils and other treatments to enhance the leather’s aesthetics, functionality and handle. The final product of the tanning and drying process is known as “crust leather” (Monira and Mostafa 2023). After undergoing finishing treatments, the leather becomes the “finished leather” ready for crafting into various products.

Even though all the materials that compose modern footwear may contribute to the deterioration of the environment, leather materials, despite their natural origin, are the ones that can have the most severe environmental impacts due to the use of chromium as a tanning agent. Through its use, it is possible to obtain more versatile, hydrothermally stable, better dyeable and soft leather. However, chrome tanning is considered to be the most polluting unit process since it releases trivalent chromium ions Cr(III) in water courses that can be further oxidized to Cr(VI) (China et al. 2020; Kanagaraj et al. 2008). As about 90% of global leather is currently produced through chromium tanning, its shavings alone contribute around 75% of solid wastes generated from leather manufacturing (da Silva et al. 2011; Erdem 2006). Since their destination are the landfills, toxic chemicals can be leached out into surrounding environments, affecting the ecosystem and human health (China et al. 2020; Van Rensburg et al. 2020).

It is known that, of all chromium salt used in the tanning process, only 55 to 70% is fixed in the leather, whereas the rest ends up in the effluents. Thus, several negative impacts are already known to occur due to this process in the aquatic ecosystem, such as, acute toxic effects; foaming problems on surface waters; inhibition of the nitrification process; eutrophication of water bodies or in opposition reduced photosynthetic activity and oxygenation of receiving water bodies due to sun blockage which is detrimental to aquatic microalgae and plants activity; depletion of dissolved oxygen promoting anaerobic condition and causing a putrefying odour of the water courses, all these processes adversely affecting the ecological functioning of aquatic resources (Carpenter et al. 2013; Dixit et al. 2015; Lofrano et al. 2013; Schilling et al. 2012; Verma et al. 2008). As for the terrestrial compartment, chrome tanning is responsible for soil acidification, reduced soil fertility and water salinization due to its highly saline nature (salt concentration); groundwater pollution through leaching of highly toxic chromium to the deeper layers of soil; deficiencies of some micronutrients such as zinc (Zn), copper (Cu) and iron (Fe); alteration in the structure of soil microbial communities, affecting its functions with possible enhanced growth of pathogenic microbes, in detriment of beneficial ones, and impacts on bioremediation process (Saxena et al. 2020, 2016).

Although all these effects caused by by-products of the tanning process are already known, there is a gap in the knowledge when it comes to understanding if the leather residue itself affects the biota of the terrestrial ecosystem. For this purpose, multi-species microcosm assays are particularly relevant since it is an artificial, simplified ecosystem that is used to simulate and predict the behaviour of natural communities under controlled conditions functioning as an intermediate testing step between single-species toxicity tests and field studies (Carbonell and Tarazona 2014). By using a multi-species bioassay approach, that tests more than one species at the same time and allows to test higher levels of biological organisation, it is possible to study the environmental fate and the overall effects of chemical compounds on soil organisms such as microorganisms, invertebrates and plants as well as the degradation, leaching or the potential for bioaccumulation of the chemicals (Carbonell and Tarazona 2014; Perrodin et al. 2011).

Taking all of this into account, the aim of this study was to characterize the ecotoxicological and citotoxicological properties of footwear manufacture by-products to the soil organisms through a multi-species microcosm experiment.

## Materials and methods

### Test substance

Two different residues from the footwear industry were used, provided by Centro Tecnológico do Calçado de Portugal (CTCP). The residues chosen were “Wet Blue” (WB) and “Finished Leather” (FL). As mentioned in the previous section, WB is a leather with a blue colour acquired when chromium salts are used and FL is the leather that underwent the finishing treatments, and it is ready to be used.

### Soil sampling and physical and chemical characterization

For this study, soil was collected in the agricultural field of Campos de Vairão, Faculty of Sciences of the University of Porto, to which no plant protection products have been applied for several years. The samples were then characterized in terms of physical and chemical properties, namely, pH in water and potassium chloride (KCl) suspensions, electrical conductivity (EC), water holding capacity (WHC), organic matter content (OM) and concentration of potentially toxic elements. The measurement of soil pH was made in a soil:water (1:5 m/v) and soil:KCl (1 M) (1:5 m/v) suspensions following ISO guideline 10,390 (ISO 1994), and the EC was measured in the same water suspension used for pH measurement (Davis and Freitas 1984), using a pH meter and a conductivity meter (Edge®, Hanna Instruments), respectively.

For WHC, the samples (50 g) were placed in polypropylene flasks with the bottom replaced with filter paper (previously weighed) and immersed in water for 3 h. After this period, the flasks were placed on a tray for 2 h with absorbent paper to remove excess water and then weighed to determine the mass of the saturated soil. The flaks were then left in the oven at 105 °C for 24 h, and, after cooling, the weight of dry soil was recorded.

The water content in the soil (moisture) was determined from the weight loss after drying at 105 °C for 24 h. After this step, the OM content was measured by the loss of ignition in a muffle furnace at 450 °C, for 8 h (SPAC—Soil and Plant Analysis Council, 2000).

The pseudo-total concentration of potentially toxic elements (PTE) in the soil samples prior to contamination was determined using inductively coupled plasma-mass spectrometry (ICP-MS) (Agilent 7700) after acid digestion in a heating block (DigiPREP MS, SCP Science), with a HNO_3_:HCl (3:1) mixture, following the USEPA method 3051A (Environmental Protection Agency 2007). The analysis of the extracts was performed for eight chemical elements: Cr, Fe, Ni, Cu, Zn, As, Cd and Pb. In each analytical batch, procedure blanks, and certified reference materials were included to assess the accuracy and the precision of the analytical method. Less than 10% of uncertainty was obtained for these inorganic elements, per replicate analysis of the soil. The results of the blank analysis were always below the detection limit, and recoveries of reference materials (Till 1 and ERM-CC141 LOAM SOIL) were within the certified value.

### Test organisms

For this study, two species of terrestrial organisms were chosen, namely, *Eisenia fetida* and *Brassica oleracea* var. *capitata*. The earthworms were obtained from LABRISK (Faculty of Sciences of the University of Porto) laboratory cultures, kept in plastic boxes (10–50 L) filled with a substrate composed of dry and defaunated (through two freeze–thawing cycles: 48 h at − 20 °C followed by 48 h at 65 °C) peat, sterilized horse manure, deionized water and CaCO_3_ to adjust the substrate pH (6.0 ± 0.5). The earthworms are fed every 2 weeks with oatmeal previously hydrated with deionized water. Only adult earthworms with developed clitellum and body mass between 300 and 600 mg were selected and acclimated before exposure for 7 days in plastic boxes with natural soil under controlled conditions at 20 ± 2 °C and a photoperiod of 16h^L^:8h^D^. *Brassica* seedlings were purchased from a local supplier and selected to have a similar vegetative development.

### Microcosm assay

For the microcosm assay, the test soil was not sieved (so that to be similar as possible to the field conditions) and moistened to reach 50% of its maximum water holding capacity and divided into three batches. The tested residues were added individually to two of the batches, and the third batch was used for the control condition with no residues. The soil batches were kept for 1 month at room temperature and natural photoperiod, being the humidity monitored and restored weekly.

After the stabilization period, the soil of each one of the three batches was divided into individual boxes (5 kg of dry soil) to start the microcosm test, which consisted of four treatments with three replicates each. Treatment 1 consisted of the control soil without any residue or organism added (CTL); in treatment 2, the soil did not receive any residue, but the two species of test organisms were added (C1); treatments 3 and 4 included the soils to which the “Wet Blue” (WB) and the “Finished Leather” (FL) residues were added, respectively, and to which both test species were also added. Each individual replicate included 5 kg_dw_ of soil, 150 g of the respective residue, 20 oligochaetes and three cabbage seedlings.

To register the initial fresh and dry weight, three seedlings similar in size were randomly selected of the set of seedlings bought. The microcosmos assay had a duration of 30 days, through which the humidity was monitored and re-established. After this period, leaf chlorophyl content (using SPAD chlorophyl meter) and the photosynthetic rate and stomatal conductance using an infrared gas analyzer (IRGA) were evaluated in the fresh plants which were later collected, and one plant per replicate was dried at 60 °C to record the dry biomass and, afterwards, used to analyse Cr bioaccumulation. The other two plants were frozen at − 80 °C to quantify photosynthetic parameters (total chlorophylls and carotenoids). As for the earthworms, the number of juveniles and cocoons in the soil were counted in each box as well as the number of adults which were frozen at − 20 °C and − 80 °C to analyse Cr bioaccumulation and oxidative stress biomarkers, respectively.

### Soil enzymes activity, potential nitrification, and nitrogen mineralization

For the determination of the soil’s enzymatic activity, 1 g of fresh soil was weighed into 15-mL centrifuge tubes; three blanks and three analytical replicates were prepared for each soil microcosm replicate and for each soil enzymatic parameter. Soil moisture content was also determined through weight loss after drying at 105 °C, for 24 h. The enzymatic activities were assessed using established protocols: dehydrogenase (Öhlinger 1996), acid phosphatase (Eivazi and Tabatabai 1977; Margesin 1996) arylsulphatase (Strobl and Traunmuller 1996; Tabatabai and Bremner 1969), carboximetil cellulase (CM-cellulase) (Mersi and Schinner 1996; Schinner and Mersi 1990) and urease (Kandeler and Gerber 1988; Schinner et al. 1996). Additionally, potential nitrification and nitrogen mineralization were also assessed following the methodologies of Berg and Rosswall (1985), Kandeler (1996a) and Kandeler (1996b). These biochemical measurements were adapted to a microplate reader as described by Ganilho et al. (2022) and Bouguerra et al. (2022).

### Determination of leaf chlorophyl content and gas exchange measurements

Leaf chlorophyl content was determined using a SPAD 502 PLUS (model KONICA MINOLTA) instrument. The youngest fully developed leaves of each pot were used for this determination. When the meter was clamped over leafy tissue, indexed chlorophyll content (0–99.9) reading was recorded. Each leaf SPAD value obtained was the average of three readings on the leaf margin.

Gas exchange measurements such as photosynthetic rate and stomatal conductance to H_2_O were evaluated in all plants using a LICOR Portable Photosynthesis System (Model LI-6400XT). The analyses were carried using 150 µmol of photons m^−2^ s^−1^ at 25 °C, flow of 350 µmol s^−1^ and CO_2_ referential rate of 400 µmol mol^−1^. After this evaluation, the leaves were frozen at − 80 °C to analyse the photosynthetic pigments.

### Determination of photosynthetic parameters

Total chlorophylls (*Ca* + *Cb*) and carotenoids of frozen leaf samples (ca. 200 mg) were extracted in 80% (v/v) acetone and quantified by spectrophotometry as described by Lichtenthaler (1987). The absorbance (Abs) at 470, 647 and 663 nm was recorded and the results obtained were expressed in mg g^−1^ fresh weigh (fw) using the following equations provided by Lichtenthaler (1987):

$$\text{Clorophyl a }(\text{Ca})=(12.25\times {\text{Abs}}_{663})-(2.79\times {\text{Abs}}_{647})$$

$$\text{Clorophyl b }(\text{Cb})=(21.50\times {\text{Abs}}_{647})-(5.10\times {\text{Abs}}_{663})$$

$$\text{Carotenoids}=\frac{\left(1000\times {\text{Abs}}_{470}\right)-\left(1.82\times \text{Ca}\right)-(85.02 \times \text{Cb})}{198}$$

### Alkaline comet assay

The experimental procedure for this assay was followed according to Reinecke and Reinecke (2004), Lourenço et al. (2012) and Fernandes et al. (2020). The earthworms removed from the soil were left to depurate overnight in plastic containers to eliminate the gut content. The coelomocytes (circulating immune cells that are essential to stress induced responses) were obtained and frozen at –80 °C in a solution containing 10% dimethyl sulphoxide (DMSO) and phosphate buffered saline (PBS) (1 × , pH 7.4) until analysis. Before the comet assay, the cell suspensions were placed at 37 °C to rapidly defrost, centrifuged 3 min at 380 g and washed with PBS (1 × , pH 7.4). All the referred procedure was conducted under yellow light, to minimize the DNA damage caused by UV radiation.

Microscope slides were prepared and the extruded coelomocytes of *E. fetida* were placed on top of the first layer of 1% normal melting point agarose. To solidify the agarose, the slides were placed on ice and then immersed in a lysing solution during 2 h in the dark at 4 °C. The slides were washed with cold PBS (1 × , pH 7.4) after lysis and immersed in the electrophoresis buffer for 15 min, to allow the unwinding of DNA. Immediately after, the electrophoresis was carried out for 10 min at 0.7 V cm^−1^ and 300 mA. The slides were then neutralized, washed with absolute ethanol, and left to dry in the dark at room temperature, for at least 24 h.

Visual scoring of cellular DNA damage on each slide was based on the categorization of 100 randomly selected cells. The comet-like formations were visually graded into five classes according to the scale followed by Fernandes et al. (2020). The results are expressed in arbitrary units following the multiplication of the number of observed cells (100) by the comet classification (0–4).

### Biomarker analyses of *E. fetida*

The neurotoxic stress biomarker acetylcholinesterase (AchE) and the oxidative stress biomarkers lipid peroxidation (LP) were analysed in the earthworms. To each replicate, containing five organisms, 7.5 mL of 0.1 M Phosphate buffer at pH 7.4 was added for homogenization, which was done with a tissue homogenizer (T10 basic ULTRA-TURRAX®) on ice. The samples were then centrifuged at 3900 rpm for 15 min, at 4 °C, and the supernatant was divided into several microtubes and stored at − 80 °C for posterior analysis of biomarkers and protein quantification. This methodology was adapted from the one followed by Correia et al. (2017).

Lipid peroxidation was measured by quantifying malondialdehyde (MDA) resulting from the reaction of thiobarbituric acid (TBA), according to the protocol described by Buege and Aust, (1978). The concentration of MDA was measured at 535 nm and the results are expressed in nmol MDA per mg of protein.

Acetylcholinesterase (AChE) activity was measured following the protocol described by Ellman et al. (1961). The hydrolysis of the substrate acetylthiocholine by the enzyme acetylcholinesterase, originates acetate and thiocholine. The thiocholine complexes with DTNB (dithiobisnitrobenzoate) creating a yellow-coloured compound which absorbance was measured at 412 nm. The activity of AChE was calculated as μmol per min per mg protein.

The protein concentration of each sample was determined following the method Bradford, (1976) adapted to 96 well’s microplates. Protein content was measured spectrophotometrically at a wavelength of 595 nm and expressed as mg mL^−1^.

All the parameters were measured in a spectrophotometer equipped with a microplate reader (Thermo Scientific™ Multiskan™ GO UV/Vis microplate spectrophotometer).

### Total Cr content in leather

Total chromium content in leather was analysed following the ISO protocol 17,072–2:2019 (ISO 2019). Leather samples of 1 g were weighed and placed in an Kjeldahl flask and submitted to a digestion with 10 mL to 20 mL of a ternary mixture of nitric acid, sulphuric acid and perchloric acid in a ratio of 3:1:1 and heated to boiling. The warming was stop after digestion was completed. The obtained suspension was cooled until room temperature re-dissolved in water and filter. Chromium was quantified by flame atomic absorption spectrometry (FAAS), using a Perkin Elmer PinAAcle 500 Atomic Absorption spectrometer.

### Total Cr content in soil after residue incorporation, earthworms’ tissues, and plant’s leaves

Total chromium content in the soil, earthworm’s tissues and plants’ leaves of the different treatments were analysed following the ISO protocols 1466:1995 (ISO 1995) and 54,321:2020 (ISO 2020). Samples of 0.5 to 1 g were weighed and placed in an Erlenmeyer and submitted to a pre-digestion conducted at room temperature overnight with 5 mL of HCl:HNO_3_ (3:1), followed by a digestion on hotplate for 4 h. The obtained suspension was cooled until room temperature and 1 mL hydrogen peroxide was added. Then, the suspension was heated on hotplate for 1 h, filtered using 0.45-µm filter and diluted with ultrapure water to 15 mL. Chromium was quantified by flame atomic absorption spectrometry (FAAS), using a Perkin Elmer PinAAcle 500 Atomic Absorption spectrometer.

### Statistical analysis

The statistical analysis was made using software GraphPad Prism 8, from GraphPad Software. All endpoints were expressed as mean ± standard deviation (SD). The effect of the footwear waste treatments on the different parameters analysed were evaluated using one-way ANOVA, after checking the homogeneity of variances by the Levene’s test. Whenever *p* ≤ 0.05, the post hoc Dunnett’s test was used to check for significant differences from the CTL.

## Results and discussion

### Soil physical and chemical parameters

The physical and chemical characterization of the agricultural soil selected for this experiment (Table 1) shows that the soil is slightly acidic (pH_H2O_ = 6.06), non-saline (EC = 61.57 µS cm^−1^) and with a high organic matter content (OM = 6.56%) (Tan 1996). As for the concentration of potentially toxic elements recorded in the test soil, presented in Table 2, these did not exceed the limit values defined by the law by decree 276/2009, issued by Ministério Regional do Ambiente do Ordenamento do Território e do Desenvolvimento (MAOTD 2009) on agricultural soils (5.5 < pH ≤ 7) that can potentially be fertilized with sewage sludge. Similarly, PTE total concentrations did not surpass the reference values for agriculture soils set up by the Portuguese Environmental Agency (APA - Agência Portuguesa do Ambiente 2019) for agriculture soils. This supports the use of the natural agricultural soil selected for the experiments.

**Table 1:** Physical and chemical characterization of the natural soil collected in the agricultural fields of *Campus of Vairão*, Faculty of Sciences of the University of Porto, namely pH measured in water and KCl (1 mol L^−1^), electric conductivity (EC), water holding capacity (WHC) and organic matter (OM). Results are expressed as mean plus standard deviation

Parameter	Mean	STD
pH_H2O_	6.06	0.05
pH_KCl_	4.60	0.04
EC (µS cm^−1^)	61.57	1.53
WHC (%)	34.10	0.68
OM (%)	6.56	0.00

**Table 2:** Concentration of potentially toxic elements (PTE), namely iron (Fe), nickel (Ni), lead (Pb), zinc (Zn), cadmium (Cd), arsenic (As), chromium (Cr) and copper (Cu), of the agricultural soil collected in the fields of *Campus of Vairão*, Faculty of Sciences of the University of Porto Vairão. Reference values for agricultural soils receptors of sewage sludge according to Ministério Regional do Ambiente do Ordenamento do Território e do Desenvolvimento (2009) and Portuguese Environmental Agency (APA - Agência Portuguesa do Ambiente 2019) for the same PTE are also presented

PTE (mg kg^−1^)								
	Fe	Ni	Pb	Zn	Cd	As	Cr	Cu
Agricultural soil	11,984	6	33	130	0.15	8	11	33
MAOTD, (2009)	–-	75	300	300	3	–-	200	100
APA (2019)	–-	37	45	290	1	11	67	62

The quantification of chromium (Cr), lead (Pb) and cadmium (Cd) performed in the soil samples (Table 3) showed an increase in the levels of Cr in the soil with the Finished Leather (FL) treatment, with an average of 128 ± 18 mg kg^−1^ whereas Pb concentrations varied between 31 ± 2 and 38 ± 2 mg kg^−1^. Taking this into consideration, FL residue seemed to have released higher concentrations of Cr, at least in the short term of the experience, as it was the residue with the higher Cr concentration. It is also important to stress that Cr concentration obtained initially (Table 2) for the agricultural soil characterization differed slightly from the present one (Table 3) probably due to soil heterogeneity and different extraction methods, used for the analysis; nevertheless, in both situations, Cr levels in the CTLs were lower than the limit concentrations represented in Table 2 and increased remarkably with the addition of the FL residue.

**Table 3:** Concentration of chromium (Cr), lead (Pb) and cadmium (Cd) in the soil samples collected from both the controls (CTL and C1), Wet Blue (WB), and Finished Leather (FL) treatments of the microcosmos assay as well as in the tested residues. Results are represented as mean ± standard deviation and expressed as mg kg^*−*1^

Treatment	Cr (mg kg^−1^)	Pb (mg kg^−1^)	Cd (mg kg^−1^)
CTL	4.3 ± 0.4	38 ± 2	< 0.2
C1	4.63 ± 0.06	31 ± 2	< 0.2
WB	10.5 ± 0.1	33 ± 4	< 0.2
FL	128 ± 18	35 ± 7	< 0.2
Wet Blue residue	14,838 ± 60	< 12	< 0.6
Finished Leather residue	19,319 ± 60	< 12	< 0.6

### Soil’s enzymatic activity

Soil’s enzymes can be used as indicators of the activity of the soil microbial community and its role on biogeochemical cycles, providing relevant data for the evaluation of soil health (García-Ruiz et al. 2008; Gregorich et al. 1994). Soil enzymes are important for the overall process of organic matter decomposition and nutrients’ cycling (Makoi and Ndakidemi 2008) being very sensitive to environmental perturbations, such as contamination by potentially toxic elements, which induce quick changes in their activity (Karaca et al. 2010; Madejón et al. 2001; Prakash Bansal 2019).

**Fig. 1:**
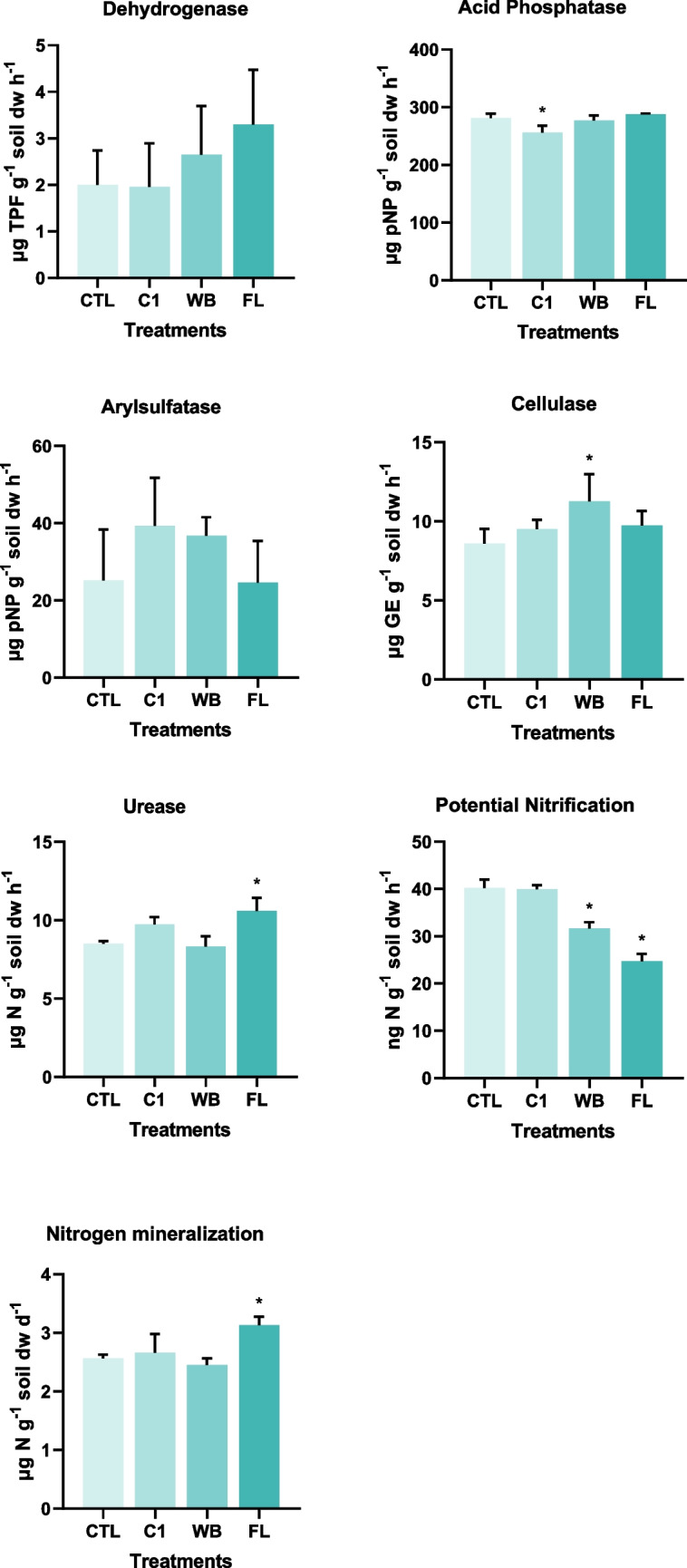
Average activity of specific soil enzymes, expressed as the amount of reaction products released by mass of soil and time unit, namely for dehydrogenase (DHA), acid phosphatase (AC), arylsulphatase (ARYL), cellulase (CEL) and urease (UREA) and of potential nitrification (PN) and nitrogen mineralization (MIN) processes in both control soils (CTL and C1) and Wet Blue (WB) and Finished Leather (FL) soil treatments. Error bars correspond to the standard deviation. Statistically significant differences compared to the control (CTL), considering *p* ≤ 0.05, are marked with a * above bars

The first interesting fact drawn from the results obtained was that, in the absence of the residues, the addition of soil organisms (such as the earthworm *E. fetida* and the plant *B. oleracea*) did not influence the soil enzymatic activity (acid phosphatase being the exception) as C1 did not show any statistically significant differences) comparing with the CTL group (that have no plants despite some earthworms were found there at the end of the assay) for either the enzymes or the microbial parameters assessed (Fig. 1). In the specific case of acid phosphatase activity, the statistically significant decrease observed in C1 could be due to the presence of earthworms in the soil. The gut of these organisms hosts many microorganisms that play a key regulatory role in the host nutrient metabolism, immune system and other physiological functions. Earthworm’s gut transit can thus be responsible for degradation and mineralization of organic matter and for the enrichment of phosphorus due to the alteration of the structure and function of the bacterial community (Wang et al. 2021). This occurs in the earthworm’s casts that are richer in several main nutrients, such as phosphorus, than the adjacent soil (Iordache, 2023). For the remaining microbial parameters, the results obtained are not in agreement with other authors that observed a positive effect on soil enzymatic activity due to the activity of earthworms (such as *E. fetida* and *E. andei*) both in contaminated soils (Wang et al. 2017; Xu et al. 2021; Zhang et al. 2021) and vermicomposting (Dume et al. 2022; Gómez-Brandón et al. 2022; Zhou et al. 2022). Even though the duration of the experiments (28–90 days) and the number of earthworms (10–50) varied between studies, the authors stated that this positive impact could be due to that fact that earthworms contribute to soil aggregate structure by burrowing, casting, mixing and aggregating soil particles into macroaggregates, which are essential for microbial activities and soil health. Moreover, earthworms can alter nutrient cycling by affecting certain enzyme activities involved in the breakdown or mineralization of N and P into inorganic forms. Nevertheless, in the case of vermicomposting, not only organisms are added but also organic matter, in a more labile form, that also influences soil enzymatic activity and microbial parameters.

Despite the changes observed mainly in microbial parameters related with the nitrogen cycle (as discussed below), no significant changes in the overall soil microbial community metabolic activity were recorded. Dehydrogenases (DHA) activity did not display significant differences between treatments. However, in the FL, there was a significant increase in the cellulase activity (*F* (3, 8) = 2.986, *p* value = 0.0960), suggesting that FL may have been a usable source for carbon added to the soil, or at least the FL residue was the one able to disturb the C:N balance of the soil, probably due to the release of dissolved elements as nitrogen compounds and, cellulases were stimulated to degrade more recalcitrant organic compounds, as cellulose. Cellulase is an important enzyme that decomposes soil organic matter into glucose, and it is also an important carbon energy source for soil microorganisms. In what regards the nitrogen biogeochemical cycle, significant differences between treatments were recorded for the urease activity (*F* (3, 8) = 10.29; *p* value = 0.0040), which was highest in the soil with the FL residues. Indeed, as these footwear residues have nitrogen in their composition (Dang et al. 2019), changes in the activity of microbial parameters related with the nitrogen cycle were expected. Urease intervenes in the nitrogen cycle in soils, and it catalyses the hydrolysis of urea into carbon dioxide and ammonia. In addition to urease, nitrogen mineralization and potential nitrification are key steps in the N cycle as the first one converts organic N in plant-available inorganic forms (Girkin and Cooper 2022) and the second is responsible for the biological oxidation of ammonium (NH_4_^+^) or ammonia (NH_3_) to nitrate (Sahrawat 2008). N mineralization followed the same trend as urease, as a statistically significant increase was observed in FL treatment in relation to the CTL soil (*F* (3,8) = 7.726, *p* value = 0.0095). Indeed, the plants that grew in the soil spiked with FL residues had higher, but not significant, growth comparing with WB treatment, which could have been caused by a higher availability of nitrogen compounds used by the plants and provided by the FL leather residues. Nevertheless, the potential nitrification was significantly inhibited in both residues treatments comparing with the control (*F* (3,8) = 81.67; *p* value < 0.0001) what was in fact not expected, at least for the FL treatment, considering that an increment in ammonium or ammonia is happening with increased mineralization and production of NH_4_^+^ in soil (Zaman et al. 1999). However, probably due to a higher capability of cabbage to absorb this nitrogen form, it was less available for nitrifiers (Baiga and Rajashekhar Rao 2017; Su et al. 2017; Turan and Sevimli 2005; Zhang et al. 2022b, a; Zhu et al. 2021). Additionally, the impact of Cr on this specific group of microorganisms cannot be neglected as both residues accounted with Cr to the soil, in particular the FL residue (Aceves et al. 2007; Buladaco et al. 2020; Visser and Parkinson 1992; Zhang et al. 2022b, a). In summary, these results suggest that each residue had a different contribution both in the soil microbial activity and in the balance of nitrogen compounds. However, from an integrated analysis, no negative effects can be inferred from the results observed.

### Microcosmos assay: effects of Cr exposure in *Brassica oleracea*

**Fig. 2:**
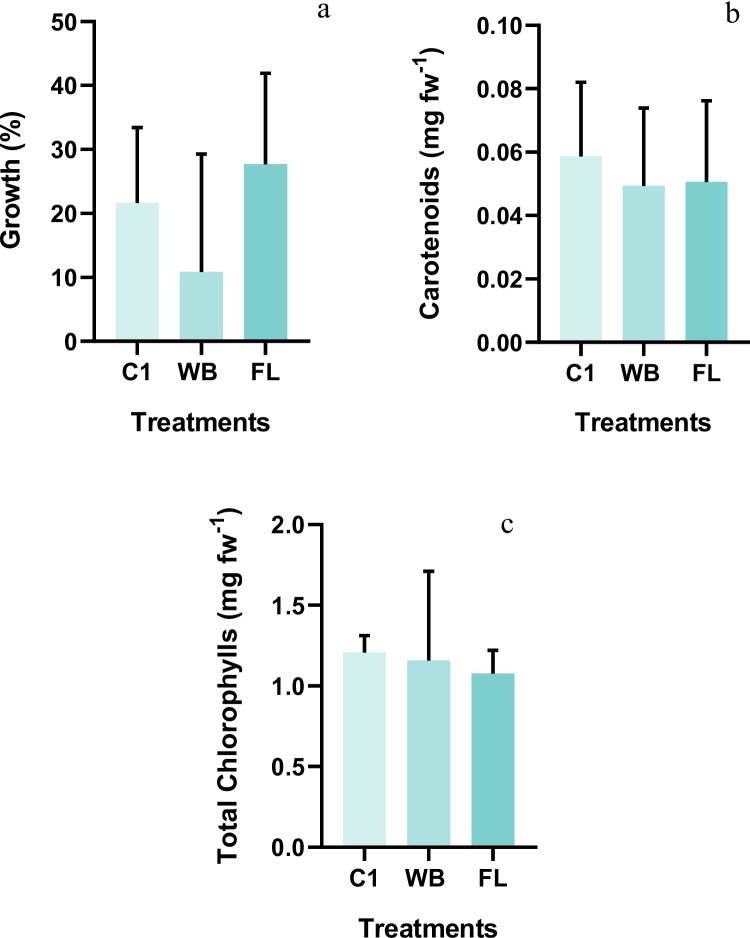
Average percentage of seedlings growth during the exposure period expressed as fresh weight (**a**), carotenoids (**b**), and total chlorophyll (**c**) content of *Brassica oleracea* plants in the control (C1), Wet Blue (WB), and Finished Leather (FL) treatments. Error bars correspond to the standard deviation

The results regarding the growth, total chlorophyll and carotenoid content in cabbage seedlings growing in different soils are described in Fig. 2 and the results of the leaf chlorophyl content measured by SPAD and photosynthetic rate and stomatal conductance are presented in Table 4. No significant statistical differences were found between the two treatments and the control group for all the parameters analysed in the cabbage. Since Cr concentration of the soil spiked with FL residues was higher than the one obtained for the soil treated with WB residue, it would be expected a decrease in plant growth that was not observed in this case. Indeed, Cr can be accumulated by plants, interfering with several metabolic processes, leading to toxicity, and causing reduced growth and biomass (Sharma et al., 1995; Rai et al. 2004). Moreover, if plants are exposed to a chromium-stressed environment, they could face a potential risk from reactive oxygen species (ROS) if the plant’s antioxidant system is not enough to counteract the negative effects of ROS overproduction (Sharma et al., 2012; Soares et al., 2019). However, it is already documented that in the tannery industry Cr is used as Cr(III) which is more strongly retained in soil particles (Bolan et al. 2003), therefore, likely not available for uptake and to impair plant growth. Further, the contribution of leather residues with nutrients (namely nitrogen) to the soil, with a possible mimicking effect of the toxicity of chromium to the plants, may not be neglected.

**Table 4:** Results obtained for the measurements with SPAD and IRGA on *Brassica oleracea* leaves in the control (CTL), Wet Blue (WB) and Finished Leather (FL) treatments

Treatment	Replica	SPAD value		Photosynthetic rate (µmol CO_2_ m^−2^ s^−1^)		Stomatal conductance (mol H_2_O m^−2^ s^−1^)	
		Mean	STD	Mean	STD	Mean	STD
C1	A	29	2	6.2	0.6	0.12	0.02
	B	30	1	6.8	0.9	0.16	0.07
	C	32	3	7.1	0.9	0.19	0.05
WB	A	32	1	5.7	0.7	0.13	0.06
	B	30	2	5.7	0.3	0.09	0.03
	C	32	2	6.5	1	0.25	0.10
FL	A	33	2	6.2	0.6	0.13	0.04
	B	30	1	5.73	0.07	0.09	0.01
	C	33	2	6.8	0.9	0.13	0.05

Carotenoids are a group of colourful pigments that play a major role in photosynthesis, the production of phytohormones and the protection of photooxidative damage (Sathasivam et al. 2021). The carotenoid content of the plants (Fig. 2b) that grew in the contaminated soil was not significantly affected by the residues, suggesting that the plants were either not under oxidative stress or that mechanisms may have been activated to mitigate it.

Regarding the total chlorophyll content (Fig. 2c), also, no significant differences were recorded between treatments. These results agreed with the ones obtained by soil–plant analyses development (SPAD) readings (Table 4). SPAD is a non-destructive tool that gives a relative measure of chlorophyll content in leaves by measuring transmittance in the red (650 nm) and infra-red (940 nm) wavelength regions (Markwell et al. 1995; Uddling et al. 2007). This device has been used in several studies to measure chlorophyll content in plants exposed to Cr either by soil contamination or by foliar application (Farid et al. 2018, 2017; Kamran et al. 2017). Negative effects of Cr contamination on plants were shown in *Ocimum tenuiflorum* L. (Rai et al. 2004), in *Triticum aestivum* L. (Ali et al. 2015), in *Zea mays* L. (Islam et al. 2016) and in *Oryza sativa* L. (Hussain et al. 2018). As reviewed by Shanker et al. (2005) and Prasad et al. (2021), Cr does not play a key role in plant metabolism and development, and has no specific transport mechanism. Still, Cr accumulation in plants is highly harmful in terms of inducing physiological and morphological alterations such as chlorosis, impaired photosynthesis through photosynthetic pigments degradation, affecting chlorophylls content and plant photosynthetic rate, which could potentially lead to plant death (Sharma et al., 1995; Rai et al. 2004). However, these effects were not recorded in this study in plants exposed to leather residues with chromium. In fact, as can be seen in Table 4, the gas exchange attributes (photosynthetic rate and stomatal conductance) of the plants that grew on the soil contaminated with WB and FL residues did not significantly differ from the ones from the control group. These parameters, which have been reported as more sensitive to exposures to soils contaminated with metals (Gavina et al. 2013) reinforced that the levels of Cr, released to the soil by the leather residues and to which plants were exposed were not phytotoxic to this crop species. In fact, as reviewed by Saud et al. (2022), most of the effects on plants reported for Cr were registered at concentrations equal or above 100 mg kg^−1^, as in mostly in soils to which Cr was added directly to the soil, thus likely more available to cause effects. This is further confirmed by the concentration of Cr in the plant’s tissues that were below the quantification limit in all the treatments (data not shown).

### Microcosmos assay: effects of Cr exposure in *Eisenia fetida*

The results of the microcosm assay regarding the terrestrial species *Eisenia fetida* (Table 5) show that in CTL, where no residue or terrestrial organisms were added, earthworms were found in those soils as well as juveniles in one of the replicas. This happened because the top of the boxes was not covered, and some earthworms avoided the replicates of the other treatments. The average number of cocoons and juveniles was higher in C1 compared to both leather treatments; however, no significant statistical differences were found. This demonstrated that the reproduction of earthworms was affected by variables other than the leather residues. In fact, similarly to the plant’s, the concentration of Cr in the earthworm’s tissues were below the quantification limit (data not shown).

**Table 5:** Results obtained for the microcosmos assay concerning the terrestrial species *Eisenia fetida* in both controls (CTL and C1), Wet Blue (WB) and Finished Leather (FL) treatments

Treatment	Replica	Initial number	Initial weight (g)	Final number	Final weight (g)	Number of cocoons	Number of juveniles
CTL	A	0	-	1	-	-	-
	B	0	-	5	-	-	2
	C	0	-	3	-	-	-
C1	A	20	7.06	20	5.35	5	6
	B	20	6.76	29	7.85	7	7
	C	20	7.01	11	2.97	9	7
WB	A	20	8.05	13	3.68	3	2
	B	20	7.29	17	4.61	5	9
	C	20	7.31	11	2.80	2	4
FL	A	20	7.26	22	6.26	8	2
	B	20	7.34	15	3.63	1	3
	C	20	7.17	18	4.91	4	1

**Fig. 3:**
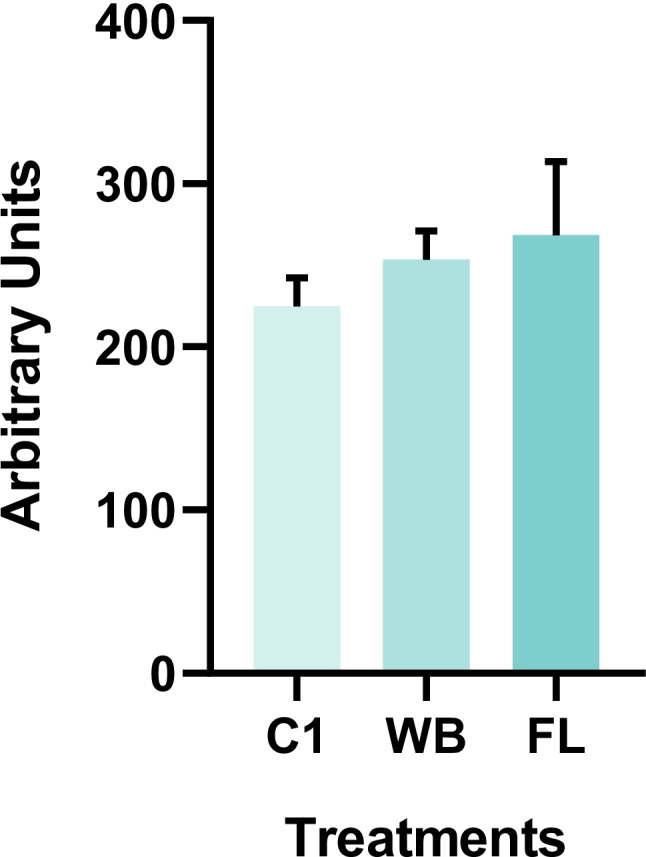
Average DNA damage in arbitrary units in *Eisenia fetida* in the control (C1), Wet Blue (WB) and Finished Leather (FL) treatments. Data is shown as average ± standard deviation

Regarding the alkaline comet assay (Fig. 3), no significant statistical differences were found between the control group and the two treatments. DNA damage under Cr exposure was reported by Bigorgne et al. (2010) in their study on genotoxic effects of Cr (491 mg kg^−1^) on *Eisenia fetida* after 2 and 4 days of exposure. However, in addition to the high Cr concentration, the exposure was performed by applying a solution of chromium nitrate directly to both artificial standardized OECD substrate and a natural soil.

**Fig. 4:**
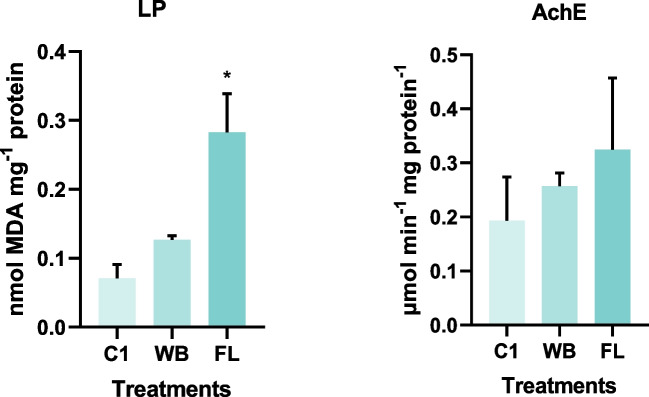
Average lipid peroxidation (LP) and acetylcholinesterase (AchE) activity in the earthworm *E. fetida* in the control (C1), Wet Blue (WB) and Finished Leather (FL) treatments. Error bars correspond to the standard deviation. Statistically significant differences compared to the control (CTL), considering *p* ≤ 0.05, are marked with a * above bars

When living organisms are exposed to environmental stresses, a biochemical response is triggered acting as early warnings. This response can be the production of reactive oxygen species (ROS), including free radicals, hydrogen peroxide, and singlet oxygen that, when in excess, can lead to oxidative stress, lipid peroxidation and cell death (Zhang et al. 2013). Lipid peroxidation levels of *E. fetida* (Fig. 4) were statistically significantly higher in FL treatment compared with the CTL group (*F* (2, 3) = 20.17; *p* value = 0.0182). Indeed, as can be seen in Table 3, soil contaminated with FL residues was the one with the highest Cr concentration, reaching 128.5 mg kg^−1^, a much higher concentration compared with the soil spiked with WB residue (10.5 mg kg^−1^). In a study conducted by Gao et al. (2016) where *E. fetida* were exposed to Cr for 1, 3, 7 and 14 days and to 0.1, 1, 10, 50 and 75 mg kg^−1^, the authors found an increase in malondialdehyde levels in the whole body, which is the major product of LP and an important indicator of ROS peroxidation, with exposure time and Cr concentration. The same response was also observed by Dzul-Caamal et al. (2020) in their study on the distribution of metals in crop soils from an agricultural region of the Yucatan Peninsula. The authors also recorded different biochemical changes in *E. fetida*, in those soils where Cr concentrations reached 88 ± 36 μg g^−1^; however, these changes may also be due to other potentially toxic elements in the soil such as Cu and Zn.

Acetylcholinesterase is the main cholinesterase in earthworms, and it plays a critical role in the functioning of the nervous system. As a biomarker of neurotoxicity it degrades acetylcholine removing it from the synaptic clefts, thus allowing the continuation of the flux of nervous impulses (Chen et al. 2020; Pereira et al. 2010). Even though no statistically significant effects were observed between the two treatments and the control group (Fig. 4), it is possible to see not an inhibition but an apparent increase of this biomarker activity in the FL treatment. In a study conducted by Rodríguez-Seijo et al. (2020) on chemical availability versus bioavailability of potentially toxic elements in mining and quarry soils, soils contaminated with 172 ± 110 mg kg^−1^ and 2604 ± 38 mg kg^−1^ of Cr significantly inhibited AchE activity in *E. fetida*. Therefore, the levels of Cr added to the soil in this study were not sufficient to promote this neurotoxic effect.

## Conclusion

With this study, it was possible to understand if the addition of footwear residues (FL and WB) to a natural soil would have consequences in terms of its soil microbial activity and non-target organisms. The use of multispecies tests as the one used in this study allows to better infer direct effects from wastes, being the effects resulting from the interaction between organisms also depicted. The Cr released by this waste may have affected some specific groups of microorganisms (e.g., nitrifiers). However, it did not affect the overall soil microbial metabolic activity, at least for the time duration of the experiment. All the results point to the higher capacity of the FL residue to release Cr and other organic compounds into the soil, which could be due to the type of treatment applied to the leather. As the footwear industry is responsible for generating large amounts of waste each year, the incorporation of its residues, into soils, in a circular economy perspective, will account for making this industry a more sustainable one. However, integrated evaluations such as the one performed in this study must be implemented, and particular attention should be given to the potential of these residues to release both organic and PTE to the soils during the degradation process, affecting the overall soil health. From this study, it is possible to conclude that some Cr-based treatments of leather increase the environmental impacts of their wastes, as was shown for the FL residue, as it displayed a higher potential to release Cr, rapidly contributing to levels not acceptable for agriculture soil use, especially if we consider possible cumulative amendments. Due to this, FL residue was also able to disturb the soil microbial community and the biogeochemical cycle of nitrogen.

From the microcosm assay performed, it was gathered that in the case of *B. oleracea*, the residues did not significantly affect its growth, total chlorophyll and carotenoid content as well as the gas exchange attributes analysed (photosynthetic rate and stomatal conductance). On the other hand, *E. fetida* earthworms were the most sensitive test organism being negatively affected, first by avoiding the treatments with FL and WB residues. Afterwards, based on the organisms that persisted in the soils, those exposed to the FL residue showed higher levels of lipid peroxidation, an indicator of oxidative stress. This was probably caused by the highest contribution of this leather residue to the amount of Cr in the soil when compared with the WB residue. Despite that, the level of oxidative stress recorded was not sufficient to induce significant DNA damage in these soil invertebrates. The FL residue also seemed to have a greater contribution with nitrogen forms to the soil that can be used by plants, accounting for their growth. In summary, by integrating organisms/parameters from three different trophic levels, we can have a better perception of the impacts of wastes from the footwear industry in the soil and in its biological processes, through a weight of evidence approach. These multispecies assays should be recommended to assess the risks of these wastes, and whenever as possible they should include other species analyse other biomarkers, to reduce the uncertainty of the evaluations.

## Data Availability

All data supporting the findings of this study are available within the paper.

## References

[CR1] Aceves MB, Velásquez RO, Vázquez RR (2007) Effects of Cr_3_^+^, Cr_6_^+^ and tannery sludge on C and N mineralization and microbial activity in semi-arid soils. J Hazard Mater 143:522–531. 10.1016/j.jhazmat.2006.09.095 17110026 10.1016/j.jhazmat.2006.09.095

[CR2] Ali S, Bharwana SA, Rizwan M, Farid M, Kanwal S, Ali Q, Ibrahim M, Gill RA, Khan MD (2015) Fulvic acid mediates chromium (Cr) tolerance in wheat (*Triticum aestivum* L.) through lowering of Cr uptake and improved antioxidant defense system. Environ Sci Pollut Res 22:10601–10609. 10.1007/s11356-015-4271-7 10.1007/s11356-015-4271-7 25744818

[CR3] APA - Agência Portuguesa do Ambiente, 2019. Solos Contaminados - Guia Técnico: Valores de Referência para Solos [WWW Document]. APA - Agência Port. do Ambient. URL https://apambiente.pt/avaliacao-e-gestao-ambiental/guias-tecnicos-0 (accessed 9.3.20).

[CR4] APICCAPS, 2020. O Calçado no Mundo - Panorama Estatístico 2020 [WWW Document]. APICCAPS - Assoc. Port. Ind. Calçado Componentes Artig. Pele Sucedâneos. URL https://www.apiccaps.pt/library/media_uploads/o-calcado-no-mundo-panorama-estatistico-2020.pdf (accessed 1.5.22).

[CR5] APICCAPS, 2022. O Calçado no Mundo - Panorama Estatístico 2022 [WWW Document]. APICCAPS - Assoc. Port. Ind. Calçado Componentes Artig. Pele Sucedâneos. URL https://www.apiccaps.pt/library/media_uploads/o-calcado-no-mundo-panorama-estatistico-2022.pdf (accessed 11.28.23).

[CR6] APICCAPS, 2023. Facts & Numbers 2023 [WWW Document]. APICCAPS - Assoc. Port. Ind. Calçado Componentes Artig. Pele Sucedâneos. URL https://www.apiccaps.pt/library/media_uploads/facts-and-numbers-2023-preview.pdf (accessed 11.28.23).

[CR7] Baiga R, Rajashekhar Rao BK (2017) Effects of biochar, urea and their co-application on nitrogen mineralization in soil and growth of Chinese cabbage. Soil Use Manag 33:54–61. 10.1111/sum.12328

[CR8] Berg P, Rosswall T (1985) Ammonium oxidizer numbers, potential and actual oxidation rates in two Swedish arable soils. Biol Fertil Soils 1:131–140. 10.1007/BF00301780

[CR9] Bigorgne E, Cossu-Leguille C, Bonnard M, Nahmani J (2010) Genotoxic effects of nickel, trivalent and hexavalent chromium on the *Eisenia fetida* earthworm. Chemosphere 80:1109–1112. 10.1016/j.chemosphere.2010.05.039 20561668 10.1016/j.chemosphere.2010.05.039

[CR10] Bolan NS, Adriano DC, Natesan R (2003) Effects of organic amendments on the reduction and phytoavailability of chromate in mineral soil. J Environ Qual 32:120–128 12549550 10.2134/jeq2003.1200

[CR11] Bouguerra S, Gavina A, Natal-da-Luz T, Sousa JP, Ksibi M, Pereira R (2022) The use of soil enzymes activity, microbial biomass, and basal respiration to assess the effects of cobalt oxide nanomaterial in soil microbiota. Appl Soil Ecol 169:104246. 10.1016/j.apsoil.2021.104246

[CR12] Bradford MM (1976) A rapid and sensitive method for the quantification of microgram quantities of protein utilizing the principle of protein-dye binding. Anal Biochem 72:248–254. 10.1016/0003-2697(76)90527-3 942051 10.1016/0003-2697(76)90527-3

[CR13] Buege, J.A., Aust, S.D., 1978. Microsomal lipid peroxidation, in: Methods in Enzymology. Academic Press, pp. 302–310. /10.1016/S0076-6879(78)52032-6 10.1016/s0076-6879(78)52032-6 672633

[CR14] Buladaco MS, Navarro DAG, Sanchez PB, Medina SM (2020) Ecotoxicological effects of chromium (VI) on seedling growth, soil nitrification and earthworm behavior. J Int Soc Southeast Asian Agric Sci 26:122–132

[CR15] Carbonell, G., Tarazona, J. V., 2014. Terrestrial Microcosms and Multispecies Soil Systems. Encycl. Toxicol. Third Ed. 486–489. 10.1016/B978-0-12-386454-3.00581-9

[CR16] Carpenter J, Sharma S, Sharma AK, Verma S (2013) Adsorption of dye by using the solid waste from leather industry as an adsorbent. Int J Eng Sci Invent 2:64–69

[CR17] Chen Y, Liu X, Leng Y, Wang J (2020) Defense responses in earthworms (*Eisenia fetida*) exposed to low-density polyethylene microplastics in soils. Ecotoxicol. Environ. Saf. 187:6. 10.1016/j.ecoenv.2019.109788 10.1016/j.ecoenv.2019.109788 31648073

[CR18] China CR, Maguta MM, Nyandoro SS, Hilonga A, Kanth SV, Njau KN (2020) Alternative tanning technologies and their suitability in curbing environmental pollution from the leather industry: a comprehensive review. Chemosphere 254:18. 10.1016/j.chemosphere.2020.126804 10.1016/j.chemosphere.2020.126804 32339802

[CR19] Concord, D., 2023. How leather is made – the tanning process [WWW Document]. Lib. Leather Goods. URL https://www.libertyleathergoods.com/how-leather-is-made/ (accessed 8.21.24).

[CR20] Correia B, Lourenço J, Marques S, Nogueira V, Gavina A, da Graça Rasteiro M, Antunes F, Mendo S, Pereira R (2017) Oxidative stress and genotoxicity of an organic and an inorganic nanomaterial to *Eisenia andrei*: SDS/DDAB nano-vesicles and titanium silicon oxide. Ecotoxicol Environ Saf 140:198–205. 10.1016/j.ecoenv.2017.02.035 28260685 10.1016/j.ecoenv.2017.02.035

[CR21] CTCP, 2012. Contribuições para a Gestão de resíduos de Couro Curtido com Crómio da indústria do Calçado [WWW Document]. CTCP - Cent. Tecnológico do Calçado Port. URL https://www.ctcp.pt/getfile/download.aspx?s=g&f=373ca0.pdf&i=395&token=Y3RjcHwyMDE5fDEw (accessed 1.5.22).

[CR22] da Silva LID, Pontes FVM, Carneiro MC, Monteiro MIC, de Almeida MD, Neto AA (2011) Evaluation of the chromium bioavailability in tanned leather shavings using the SM&T sequential extractions scheme. Chem. Speciat. Bioavailab. 23:183–187. 10.3184/095422911X13027118597382

[CR23] Dang X, Yang M, Zhang B, Chen H, Wang Y (2019) Recovery and utilization of collagen protein powder extracted from chromium leather scrap waste. Environ Sci Pollut Res 26:7277–7283. 10.1007/s11356-019-04226-x 10.1007/s11356-019-04226-x 30684174

[CR24] Davis, J., Freitas, F., 1984. Physical and chemical methods of soil and water analysis. FAO Soils Bull. no.10.

[CR25] Dixit S, Yadav A, Dwivedi PD, Das M (2015) Toxic hazards of leather industry and technologies to combat threat: a review. J Clean Prod 87:39–49. 10.1016/j.jclepro.2014.10.017

[CR26] Dume, B., Hanc, A., Svehla, P., Michal, P., Solcova, O., Chane, D., Nigussie, A., 2022. Nutrient recovery and changes in enzyme activity during vermicomposting of hydrolysed chicken feather residue. Environ. Technol. 1–15. 10.1080/09593330.2022.2147451 10.1080/09593330.2022.2147451 36368925

[CR27] Dzul-Caamal R, Vega-López A, von Osten JR (2020) Distribution of heavy metals in crop soils from an agricultural region of the Yucatan Peninsula and biochemical changes in earthworm *Eisenia foetida* exposed experimentally. Environ Monit Assess. 10.1007/s10661-020-08273-7 10.1007/s10661-020-08273-7 32382918

[CR28] Eivazi F, Tabatabai MA (1977) Phosphatases in soils. Soil Biol Biochem 9:167–172. 10.1016/0038-0717(77)90070-0

[CR29] Ellman GL, Courtney KD, Andres V, Featherstone RM (1961) A new and rapid colorimetric determination of acetylcholinesterase activity. Biochem Pharmacol 7:88–95. 10.1016/0006-2952(61)90145-9 13726518 10.1016/0006-2952(61)90145-9

[CR30] Environmental Protection Agency, 2007. Method 3051A: microwave assisted acid digestion of sediments, sludges, soils, and oils [WWW Document]. EPA - Environ. Prot. Agency. URL https://www.epa.gov/sites/production/files/2015-12/documents/3051a.pdf (accessed 9.3.20).

[CR31] Erdem M (2006) Chromium recovery from chrome shaving generated in tanning process. J Hazard Mater 129:143–146. 10.1016/j.jhazmat.2005.08.021 16202513 10.1016/j.jhazmat.2005.08.021

[CR32] European Commission, 2020. Demonstration of a new business and consumption model for the circular economy in the footwear sector [WWW Document]. Eur. Comm. URL https://webgate.ec.europa.eu/life/publicWebsite/project/LIFE19-ENV-ES-000118/demonstration-of-a-new-business-and-consumption-model-for-the-circular-economy-in-the-footwear-sector (accessed 1.21.22).

[CR33] Farid M, Ali S, Akram NA, Rizwan M, Abbas F, Bukhari SAH, Saeed R (2017) Phyto-management of Cr-contaminated soils by sunflower hybrids: physiological and biochemical response and metal extractability under Cr stress. Environ Sci Pollut Res 24:16845–16859. 10.1007/s11356-017-9247-3 10.1007/s11356-017-9247-3 28573560

[CR34] Farid M, Ali S, Rizwan M, Ali Q, Saeed R, Nasir T, Abbasi GH, Rehmani MIA, Ata-Ul-Karim ST, Bukhari SAH, Ahmad T (2018) Phyto-management of chromium contaminated soils through sunflower under exogenously applied 5-aminolevulinic acid. Ecotoxicol Environ Saf 151:255–265. 10.1016/j.ecoenv.2018.01.017 29353175 10.1016/j.ecoenv.2018.01.017

[CR35] Fernandes S, Nogueira V, Lourenço J, Mendo S, Pereira R (2020) Inter-species bystander effect: *Eisenia fetida* and *Enchytraeus albidus* exposed to uranium and cadmium. J Hazard Mater 399:122972. 10.1016/j.jhazmat.2020.122972 32526440 10.1016/j.jhazmat.2020.122972

[CR36] Ganilho C, da Silva MB, Paiva C, de Menezes TI, dos Santos MR, Pereira CM, Pereira R, Andreani T (2022) Environmental safety assessments of lipid nanoparticles loaded with lambda-cyhalothrin. Nanomaterials 12:1–20. 10.3390/nano12152576 10.3390/nano12152576 PMC9370418 35957012

[CR37] Gao C, Xu J, Li J, Liu Z (2016) Determination of metallothionein, malondialdehyde, and antioxidant enzymes in earthworms (*Eisenia fetida*) following exposure to chromium. Anal Lett 49:1748–1757. 10.1080/00032719.2015.1120738

[CR38] García-Ruiz R, Ochoa V, Hinojosa MB, Carreira JA (2008) Suitability of enzyme activities for the monitoring of soil quality improvement in organic agricultural systems. Soil Biol Biochem 40:2137–2145. 10.1016/j.soilbio.2008.03.023

[CR39] Gavina A, Antunes SC, Pinto G, Claro MT, Santos C, Gonçalves F, Pereira R (2013) Can physiological endpoints improve the sensitivity of assays with plants in the risk assessment of contaminated soils? PLoS One 8:1–12. 10.1371/journal.pone.0059748 10.1371/journal.pone.0059748 PMC3615127 23565165

[CR40] Girkin, N.T., Cooper, H. V., 2022. Nitrogen and ammonia in soils. Ref. Modul. Earth Syst. Environ. Sci. 142–151. 10.1016/B978-0-12-822974-3.00010-0

[CR41] Gómez-Brandón M, Fornasier F, de Andrade N, Domínguez J (2022) Influence of earthworms on the microbial properties and extracellular enzyme activities during vermicomposting of raw and distilled grape marc. J. Environ. Manage. 319:1–9. 10.1016/j.jenvman.2022.115654 10.1016/j.jenvman.2022.115654 35792389

[CR42] Gregorich EG, Carter MR, Angers DA, Monreal CM, Ellert BH (1994) Towards a minimum data set to assess soil organic matter quality in agricultural soils. Can J Soil Sci 74:367–385. 10.4141/cjss94-051

[CR43] Hussain A, Ali S, Rizwan M, Ziaur Rehman M, Hameed A, Hafeez F, Alamri SA, Alyemeni MN, Wijaya L (2018) Role of zinc–lysine on growth and chromium uptake in rice plants under Cr stress. J Plant Growth Regul 37:1413–1422. 10.1007/s00344-018-9831-x

[CR44] Iordache M (2023) Chemical composition of earthworm casts as a tool in understanding the earthworm contribution to ecosystem sustainability – a review. Plant Soil Environ 69:247–268. 10.17221/461/2022-PSE

[CR45] Islam F, Yasmeen T, Arif MS, Riaz M, Shahzad SM, Imran Q, Ali I (2016) Combined ability of chromium (Cr) tolerant plant growth promoting bacteria (PGPB) and salicylic acid (SA) in attenuation of chromium stress in maize plants. Plant Physiol Biochem 108:456–467. 10.1016/j.plaphy.2016.08.014 27575042 10.1016/j.plaphy.2016.08.014

[CR46] ISO, 1994. ISO 10390:1994. Soil quality: determination of pH. International Organization for Standardization. Geneva, Switzerland.

[CR47] ISO, 1995. ISO 11466:1995. Soil quality — extraction of trace elements soluble in aqua regia. Geneva, Switzerland.

[CR48] ISO, 2019. ISO 17072–2:2019. Leather - chemical determination of metal content. Part 2: Total metal content. Geneva, Switzerland.

[CR49] ISO, 2020. ISO 54321:2020. Soil, treated biowaste, sludge and waste — digestion of aqua regia soluble fractions of elements. Geneva, Switzerland.

[CR50] Kamran MA, Bibi S, Xu R. Kou, Hussain S, Mehmood K, Chaudhary HJ (2017) Phyto-extraction of chromium and influence of plant growth promoting bacteria to enhance plant growth. J. Geochemical Explor. 182:269–274. 10.1016/j.gexplo.2016.09.005

[CR51] Kanagaraj J, Chandra Babu NK, Mandal AB (2008) Recovery and reuse of chromium from chrome tanning waste water aiming towards zero discharge of pollution. J Clean Prod 16:1807–1813. 10.1016/j.jclepro.2007.12.005

[CR52] Kandeler E, Gerber H (1988) Short-term assay of soil urease activity using colorimetric determination of ammonium. Biol Fertil Soils 6:68–72

[CR53] Kandeler, E., 1996a. Nitrification and denitrification, in: Schinner, F., Öhlinger, R., Kandeler, E., Margesin, R. (Eds.), Methods in Soil Biology. Springer-Verlag Berlin Heidelberg, New York, pp. 146–148.

[CR54] Kandeler, E., 1996b. Nitrogen mineralization, in: Schinner, F., Öhlinger, R., Kandeler, E., Margesin, R. (Eds.), Methods in soil biology. Springer-Verlag Berlin Heidelberg, New York, pp. 135–143.

[CR55] Karaca, A., Cetin, S.C., Turgay, O.C., Kizilkaya, R., 2010. Effects of heavy metals on soil enzyme activities, in: Sherameti, I., Varma, A. (Eds.), Soil Biology. Springer-Verlag Berlin Heidelberg, p. 27. 10.1007/978-3-642-02436-8

[CR56] Lichtenthaler HK (1987) Chlorophylls and carotenoids: pigments of photosynthetic biomembranes. Methods Enzymol 148:350–382. 10.1016/0076-6879(87)48036-1

[CR57] Lofrano G, Meriç S, Zengin GE, Orhon D (2013) Chemical and biological treatment technologies for leather tannery chemicals and wastewaters: a review. Sci Total Environ 461–462:265–281. 10.1016/j.scitotenv.2013.05.004 10.1016/j.scitotenv.2013.05.004 23735721

[CR58] Lourenço J, Pereira R, Silva A, Carvalho F, Oliveira J, Malta M, Paiva A, Gonçalves F, Mendo S (2012) Evaluation of the sensitivity of genotoxicity and cytotoxicity endpoints in earthworms exposed in situ to uranium mining wastes. Ecotoxicol Environ Saf 75:46–54. 10.1016/j.ecoenv.2011.08.024 21955884 10.1016/j.ecoenv.2011.08.024

[CR59] Madejón E, Burgos P, López R, Cabrera F (2001) Soil enzymatic response to addition of heavy metals with organic residues. Biol Fertil Soils 34:144–150. 10.1007/s003740100379

[CR60] Makoi JHJR, Ndakidemi PA (2008) Selected soil enzymes: examples of their potential roles in the ecosystem. Afr J Biotechnol 7:181–191. 10.5897/AJB07.590

[CR61] Margesin R (1996) Enzymes involved in phosphorus metabolism. In: Schinner F, Öhlinger R, Kandeler E, Margesin R (eds) Methods in soil biology. Springer-Verlag, Berlin Heidelberg, New York, pp 213–216

[CR62] Markwell J, Osterman JC, Mitchell JL (1995) Calibration of the Minolta SPAD-502 leaf chlorophyll meter. Photosynth Res 46:467–472. 10.1007/BF00032301 10.1007/BF00032301 24301641

[CR63] von Mersi W, Schinner F (1996) Enzymes involved in carbon metabolism. In: Schinner F, Öhlinger R, Kandeler E, Margesin R (eds) Methods in soil biology. Springer-Verlag, Berlin Heidelberg, New York, pp 190–192

[CR64] Ministério Regional do Ambiente do Ordenamento do Território e do Desenvolvimento, 2009. Decreto-Lei N.276/2009, Diário da República no 192, Série I de 2 de Outubro de 2009.

[CR65] Monira U, Mostafa MG (2023) Leather industrial effluent and environmental concerns : a review. Sustain Water Resour Manage 9:1–14. 10.1007/s40899-023-00969-1

[CR66] Öhlinger R (1996) Enzymes involved in intracellular metabolism. In: Schinner F, Öhlinger R, Kandeler E, Margesin R (eds) Methods in Soil Biology. Springer-Verlag, Berlin Heidelberg, New York, pp 241–242

[CR67] Pereira JL, Antunes SC, Ferreira AC, Gonçalves F, Pereira R (2010) Avoidance behavior of earthworms under exposure to pesticides: is it always chemosensorial? Journal of Environmental Science and Health, Part B 45:229–232. 10.1080/03601231003613625 10.1080/03601231003613625 20390955

[CR68] Perrodin Y, Boillot C, Angerville R, Donguy G, Emmanuel E (2011) Ecological risk assessment of urban and industrial systems: a review. Sci Total Environ 409:5162–5176. 10.1016/j.scitotenv.2011.08.053 21944201 10.1016/j.scitotenv.2011.08.053

[CR69] Prakash Bansal, O., 2019. The influence of potentially toxic elements on soil biological and chemical properties, in: Metals in Soil - Contamination and Remediation. pp. 1–14. 10.5772/intechopen.81348

[CR70] Prasad S, Kumar K, Kumar S, Gupta N, Marina M, Cabral-pinto S, Rezania S, Radwan N, Alam J (2021) Chromium contamination and effect on environmental health and its remediation : a sustainable approaches. J. Environ. Manage. 285:1–22. 10.1016/j.jenvman.2021.112174 10.1016/j.jenvman.2021.112174 33607566

[CR71] Rai V, Vajpayee P, Singh SN, Mehrotra S (2004) Effect of chromium accumulation on photosynthetic pigments, oxidative stress defense system, nitrate reduction, proline level and eugenol content of *Ocimum tenuiflorum* L. Plant Sci. 167:1159–1169. 10.1016/j.plantsci.2004.06.016

[CR72] Reinecke SA, Reinecke AJ (2004) The comet assay as biomarker of heavy metal genotoxicity in earthworms. Arch Environ Contam Toxicol 46:208–215. 10.1007/s00244-003-2253-0 15106672 10.1007/s00244-003-2253-0

[CR73] Rodríguez-Seijo A, Lourenço J, Arenas-Lago D, Mendo S, Vega FA, Pereira R (2020) Chemical availability versus bioavailability of potentially toxic elements in mining and quarry soils. Chemosphere 251:1–9. 10.1016/j.chemosphere.2020.126421 10.1016/j.chemosphere.2020.126421 32443230

[CR74] Sahrawat KL (2008) Factors affecting nitrification in soils. Commun Soil Sci Plant Anal 39:1436–1446. 10.1080/00103620802004235

[CR75] Samidurai S, Khambhaty Y, Selvi T (2022) Bio - preservation of raw hides / skins: a review on greener substitute to conventional salt curing. Environ Sci Pollut Res 29:64513–64535. 10.1007/s11356-022-22027-7 10.1007/s11356-022-22027-7 35867302

[CR76] Sathasivam R, Radhakrishnan R, Kim JK, Park SU (2021) An update on biosynthesis and regulation of carotenoids in plants. South Afr J Bot 140:290–302. 10.1016/j.sajb.2020.05.015

[CR77] Saud S, Wang D, Fahad S, Javed T, Jaremko M, Abdelsalam NR, Ghareeb RY (2022) The impact of chromium ion stress on plant growth, developmental physiology, and molecular regulation. Front Plant Sci 13:1–16. 10.3389/fpls.2022.994785 10.3389/fpls.2022.994785 PMC9651928 36388512

[CR78] Saxena, G., Chandra, R., Bharagava, R.N., 2016. Environmental pollution, toxicity profile and treatment approaches for tannery wastewater and its chemical pollutants, in: de Voogt, P. (Ed.), Reviews of environmental contamination and toxicology. Springer, Cham, pp. 31–69. 10.1007/398 10.1007/398_2015_5009 26795766

[CR79] Saxena, G., Purchase, D., Bharagava, R.N., 2020. Bioremediation of industrial waste for environmental safety, in: Saxena, G., Bharagava, R. (Eds.), Bioremediation of Industrial Waste for Environmental Safety. Springer Singapore, pp. 381–398. 10.1007/978-981-13-1891-7

[CR80] Schilling K, Bletterie U, Kroiss H, Zessner M (2012) Adapting the Austrian edict on wastewater emissions for tanneries as consequence of foam formation on surface waters. Environ Sci Policy 23:68–73. 10.1016/j.envsci.2012.07.021

[CR81] Schinner, F., Ohlinger, R., Kandeler, E., Margesin, R., 1996. Methods in soil biology, 1st ed. Springer-Verlag Berlin Heidelberg, New York. 10.1007/978-3-642-60966-4

[CR82] Schinner F, von Mersi W (1990) Xylanase-, CM-cellulase- and invertase activity in soil: an improved method. Soil Biol Biochem 22:511–515. 10.1016/0038-0717(90)90187-5

[CR83] Shanker AK, Cervantes C, Loza-tavera H, Avudainayagam S (2005) Chromium toxicity in plants. Environ. Int. 31:739–753. 10.1016/j.envint.2005.02.003 15878200 10.1016/j.envint.2005.02.003

[CR84] Sharma DC, Chatterjee C, Sharma CP (1995) Chromium accumulation and its effects on wheat (Triticum aestivum L. cv. HD 2204) metabolism. Plant Sci 111:145–151. 10.1016/0168-9452(95)04230-R

[CR85] Sharma P, Jha AB, Dubey RS, Pessarakli M (2012) Reactive oxygen species, oxidative damage, and antioxidative defense mechanism in plants under stressful conditions. J Bot 2012:1–26. 10.1155/2012/217037

[CR86] Soares C, Carvalho MEA, Azevedo RA, Fidalgo F (2019) Plants facing oxidative challenges—a little help from the antioxidant networks. Environ Exp Bot 161:4–25. 10.1016/j.envexpbot.2018.12.009

[CR87] SPAC - Soil and Plant Analysis Council, 2000. Soil analysis handbook of reference methods, 1st ed. CRC, Boca Raton, Florida. 10.1201/9780203739433

[CR88] Strobl W, Traunmuller M (1996) Enzymes involved in sulfur metabolism. In: Schinner F, Öhlinger R, Kandeler E, Margesin R (eds) Methods in Soil Biology. Springer-Verlag, Berlin Heidelberg, New York, pp 230–231

[CR89] Su JC, He ZQ, Li J, Lu J, Yu JH, Zhang GB (2017) Effect of combined application of ammonium and nitrate on nutrient utilization and yield of mini Chinese cabbage. Agric Res Arid Areas 35:45–53. 10.7606/J.ISSN.1000-7601.2017.04.08

[CR90] Suresh V, Kanthimathi M, Thanikaivelan P, Rao JR, Nair BU (2001) An improved product-process for cleaner chrome tanning in leather processing. J Clean Prod 9:483–491. 10.1016/S0959-6526(01)00007-5

[CR91] Tabatabai MA, Bremner JM (1969) Use of p-nitrophenyl phosphate for assay of soil phosphatase activity. Soil Biol Biochem 1:301–307. 10.1016/0038-0717(69)90012-1

[CR92] Tan KH (1996) Soil sampling, preparation and analysis. Marcel Dekker Inc, New York

[CR93] Turan M, Sevimli F (2005) Influence of different nitrogen sources and levels on ion content of cabbage (*Brassica oleracea* var. capitate). New Zeal J Crop Hortic Sci 33:241–249. 10.1080/01140671.2005.9514356

[CR94] Uddling J, Gelang-Alfredsson J, Piikki K, Pleijel H (2007) Evaluating the relationship between leaf chlorophyll concentration and SPAD-502 chlorophyll meter readings. Photosynth Res 91:37–46. 10.1007/s11120-006-9077-5 10.1007/s11120-006-9077-5 17342446

[CR95] Van Rensburg ML, Nkomo SL, Mkhize NM (2020) Life cycle and end-of-life management options in the footwear industry: a review. Waste Manag Res 38:599–613. 10.1177/0734242X20908938 32181706 10.1177/0734242X20908938

[CR96] Verma T, Ramteke PW, Garg SK (2008) Quality assessment of treated tannery wastewater with special emphasis on pathogenic *E. coli* detection through serotyping. Environ Monit Assess 145:243–249. 10.1007/s10661-007-0033-4 18044007 10.1007/s10661-007-0033-4

[CR97] Visser S, Parkinson D (1992) Soil biological criteria as indicators of soil quality: soil microorganisms. Am J Altern Agric 7:33–37. 10.1017/S0889189300004434

[CR98] Wang C, Zhang Q, Wang F, Liang W (2017) Toxicological effects of dimethomorph on soil enzymatic activity and soil earthworm (*Eisenia fetida*). Chemosphere 169:316–323. 10.1016/j.chemosphere.2016.11.090 27886533 10.1016/j.chemosphere.2016.11.090

[CR99] Wang N, Wang W, Jiang Y, Dai W, Li P, Yao D, Wang J, Shi Y, Cui Z, Cao H, Dong Y, Wang H (2021) Variations in bacterial taxonomic profiles and potential functions in response to the gut transit of earthworms (Eisenia fetida) feeding on cow manure. Sci Total Environ 787:147392. 10.1016/j.scitotenv.2021.147392 10.1016/j.scitotenv.2021.147392 34000543

[CR100] Xu Z, Yang Z, Zhu T, Shu W, Geng L (2021) Ecological improvement of antimony and cadmium contaminated soil by earthworm *Eisenia fetida*: soil enzyme and microorganism diversity. Chemosphere 273:129496. 10.1016/j.chemosphere.2020.129496 33524758 10.1016/j.chemosphere.2020.129496

[CR101] Zaman M, Di HJ, Cameron KC, Frampton CM (1999) Gross nitrogen mineralization and nitrification rates and their relationships to enzyme activities and the soil microbial biomass in soils treated with dairy shed effluent and ammonium fertilizer at different water potentials. Biol Fertil Soils 29:178–186. 10.1007/s003740050542

[CR102] Zhang Q, Zhu L, Wang J, Xie H, Wang J, Han Y, Yang J (2013) Oxidative stress and lipid peroxidation in the earthworm *Eisenia fetida* induced by low doses of fomesafen. Environ Sci Pollut Res 20(1):201–208. 10.1007/s11356-012-0962-5 10.1007/s11356-012-0962-5 22585392

[CR103] Zhang Q, Li S, Saleem M, Yasir M, Xiang J (2021) Biochar and earthworms synergistically improve soil structure, microbial abundance, activities and pyraclostrobin degradation. Appl Soil Ecol 168:1–8. 10.1016/j.apsoil.2021.104154

[CR104] Zhang S, Zhang Y, Wang Y, Hao Y, Su W, Sun G, Liu H, Chen R, Song S (2022) Nitrogen absorption pattern detection and expression analysis of nitrate transporters in flowering Chinese cabbage. Horticulturae 8:1–15. 10.3390/horticulturae8030188

[CR105] Zhang X, Zhang X, Li L, Fu G, Liu X, Xing S, Feng H, Chen B (2022) The toxicity of hexavalent chromium to soil microbial processes concerning soil properties and aging time. Environ Res 204:1–12. 10.1016/j.envres.2021.111941 10.1016/j.envres.2021.111941 34474034

[CR106] Zhou Y, Li H, Guo W, Liu H, Cai M (2022) The synergistic effect between biofertility properties and biological activities in vermicomposting: a comparable study of pig manure. J Environ Manage 324:1–8. 10.1016/j.jenvman.2022.116280 10.1016/j.jenvman.2022.116280 36183526

[CR107] Zhu Y, Qi B, Hao Y, Liu H, Sun G, Chen R, Song S (2021) Appropriate NH_4_^+^ / NO_3_^−^ ratio triggers plant growth and nutrient uptake of flowering Chinese cabbage by optimizing the pH value of nutrient solution. Front Plant Sci 12:1–16. 10.3389/fpls.2021.656144 10.3389/fpls.2021.656144 PMC8121088 33995453

